# Applicability of personal laser scanning in forestry inventory

**DOI:** 10.1371/journal.pone.0211392

**Published:** 2019-02-27

**Authors:** Shilin Chen, Haiyang Liu, Zhongke Feng, Chaoyong Shen, Panpan Chen

**Affiliations:** Precision Forestry Key Laboratory of Beijing, Forestry College, Beijing Forestry University, Beijing, China; Universidade Federal de Uberlandia, BRAZIL

## Abstract

Light Detection and Ranging (LiDAR) technology has been widely used in forestry surveys in the form of airborne laser scanning (ALS), terrestrial laser scanning (TLS), and mobile laser scanning (MLS). The acquisition of important basic tree parameters (e.g., diameter at breast height and tree position) in forest inventory did not solve the problem of low measurement efficiency or weak GNSS signal under the canopy. A personal laser scanning (PLS) device combined with SLAM technology provides an effective solution for forest inventory under complex conditions with its light weight and flexible mobility. This study proposes a new method for calculating the volume of a cylinder using point cloud data obtained by a PLS device by fitting to a polygonal cylinder to calculate the diameter of the trunk. The point cloud data of tree trunks of different thickness were modeled using different fitting methods. The rate of correct tree trunk detection was 93.3% and the total deviation of the estimations of tree diameter at breast height (DBH) was -1.26 cm. The root mean square errors (RMSEs) of the estimations of the extracted DBH and the tree position were 1.58 cm and 26 cm, respectively. The survey efficiency of the personal laser scanning (PLS) device was 30m^2^/min for each investigator, compared with 0.91m^2^/min for the field survey. The test demonstrated that the PLS device combined with the SLAM algorithm provides an efficient and convenient solution for forest inventory.

## Introduction

Forest resource inventory is a key basic task of ecological construction, forestry development and forest resource management. Tree location and diameter at breast height (DBH) are the crucial parameters in forest inventory. The spatial distribution information of tree location is the fundamental parameter for the calibration of the individual-tree-based inventory, and also the main matching criterion between reference data and measured data [1]. By measuring the DBH of the trees, we can obtain the diameter distribution which describes the forest structure [2], as well as the log yield and stem quality [3]. The traditional methods, based on field inventory work for tree location calculation and DBH measurement, are labor intensive, time consuming, and limited in their spatial extent. Therefore, laser scanning technology, including airborne laser scanning (ALS), terrestrial laser scanning (TLS), and mobile laser scanning (MLS), has been widely investigated for applications in forest inventory [4–6].

Airborne laser scanning (ALS) is an effective way to retrieve biophysical variables and update forest investigation maps. Many scholars and research organizations have put tremendous efforts into developing methods for the application of ALS in forest surveying. With a strong penetrating power, the laser can penetrate the canopy, foliage, understory, etc., and obtain detailed tree parameter information, which is very important for fine modeling. Consequently, ALS is widely used in forest investigations for tree height estimation [7], stem volume estimation [8], tree crown volume estimation [9–11], tree species classification [12] and measurement of forest growth [13]. Nevertheless, the application of ALS in forest investigations largely depends on the quality and quantity of field reference data, especially with the currently applied ALS-based inventory technique, which is an area-based inventory in which field references determine the output of each raster cell based on non-parametric estimates [14]. The failure to obtain more detailed structural parameters of trees due to canopy occlusion and low density point clouds per unit area are major factors limiting the further development of airborne laser scanning in forestry surveys. In practice, high-accuracy data acquisition and appropriate measurement methods are always preferred.

Terrestrial laser scanning (TLS) has been demonstrated as an effective technique for acquiring detailed information on tree attributes in forest sample plots during the last two decades [15]. Compared with ALS, the point cloud data acquired from TLS are dense enough to determine the spatial distribution of the trees [16] and to extract almost the whole geometry of each tree with high precision. Moreover, the TLS in the plot can also easily capture the information on basic tree attributes, such as the DBH and the tree height, at the plot level [17,18]. By reconstructing the stem model, other variables of tree structure, such as stem volume, stem curvature, stem quality and biomass, can be estimated, and the results of the accuracy evaluation are comparable to the best national allometric models [19,20]. Three data acquisition approaches have been reported in TLS-based field measurements: single-scan, multi-scan and multi-single-scan. The single-scan (SS) mode uses a laser scanner that is placed at the plot center to obtain a single full field-of-view (e.g., 360°×310°) scan of point cloud data of targets in the surrounding environment. The multi-scan (MS) mode is typically implemented by placing the scanner inside and outside of the sample plot to obtain more detailed point cloud data of the sample targets. The multi-single-scan (MSS) mode combines a single-scan mode at multiple scanning sites to preform sample plot observation [19,21]. The three scan approaches can be used to acquire high-quality point cloud data. However, the single scan mode has serious occlusion problems, and the multi-scan mode is time-consuming but provides the best data sets [14].

In practice, the TLS instrument needs to be statically placed in the sample plot for scanning to acquire point cloud data, which greatly limits the mobility of data acquisition. For large-scale sample-plot investigation, this methodology is time-consuming and requires many reads to build sufficient point clouds for describing the forest environment [22]. Although the algorithms have been researched to improve the TLS point cloud precision and accuracy [18,23,24], there are still some algorithms and methods that need further improvement, such as extracting forest attributes from TLS data and data acquisition protocols [25]. These drawbacks that cannot be eliminated are still limiting the processing efficiency and accuracy of extracting forest attributes. The use of a mobile laser scanner (MLS) would reduce the drawbacks, especially in terms of occlusion and mobility [25].

A mobile laser scanner (MLS) system offers a powerful tool to solve the problem of trees occlusion and inability to move within the TLS, and greatly reduces the required time and costs. An MLS system typically combines one or several laser scanner(s) with an inertial measurement unit (IMU) and a Global Navigation Satellite System (GNSS) tracker that provides real-time position information. The data acquired by MLS is less precise than TLS point cloud data due to the propagation of positioning errors within MLS point cloud data [14]. MLS systems are predominantly mounted on vehicles for urban mapping and forest survey in flat areas [26,27]. Liang *et al*. [28,29] have conducted in-depth research on the application potential of MLS in forest inventory and the evaluation of the accuracy of extracted tree parameters. Yang *et al*. [26, 30–33] have done significant research and analysis on MLS point cloud data, developing many effective methods for extracting objects of interest from point clouds. MLS has certain terrain constraints when conducting forest surveys in the field, such as steep terrain, dense undergrowth, and barriers such as branches. In order to reduce the terrain constraints, Liang *et al*. [28] developed an MLS device mounted onto an all-terrain vehicle to conduct forest plot mapping, which has great potential for the wider application of MLS in forestry. Nevertheless, the lack of GNSS signals or weak GNSS signals under the canopy has become the greatest challenge for the application of MLS in forestry surveys [34].

Recently, Personal laser scanning (PLS), as an emerging concept, was introduced by Juha *et al*. [35], as well as Liang *et al* [14]. The introduction of PLS started in 2013, and the first system prototype was large in size and weighed approximately 30 kg, which limited its operability and mobility. To our best knowledge, the PLS equipment was first used in forest surveys in 2014 [14]. Subsequently, Ryding *et al*. [22] and Cabo *et al*. [36] also introduced PLS technology and extracted DBH and tree height information from point cloud data. PLS has the potential to improve mapping efficiency compared with conventional field measurements, and to compensate for the limitations of other laser scanning techniques, such as having to transport the scanner and associated equipment from site to site, which is one the major disadvantage of TLS, in addition to the need for certain terrain conditions and GNSS signals, which limit the application of MLS [37].

In this study, we compared the results obtained using PLS equipment with those of field measurements, and proposed a new method for calculating the DBH. The objectives of this study were (1) to estimate the accuracy of PLS point cloud data extraction DBH, tree location and trunk detection; (2) provide an effective method to solve the problem of forest inventories when there is no GNSS signal or weak GNSS signals under the forest canopy; (3) analyze the advantages and challenges of PLS; (4) explore the application potential of the PLS in forest inventory.

## Materials and methods

### Study area

**Fig 1:**
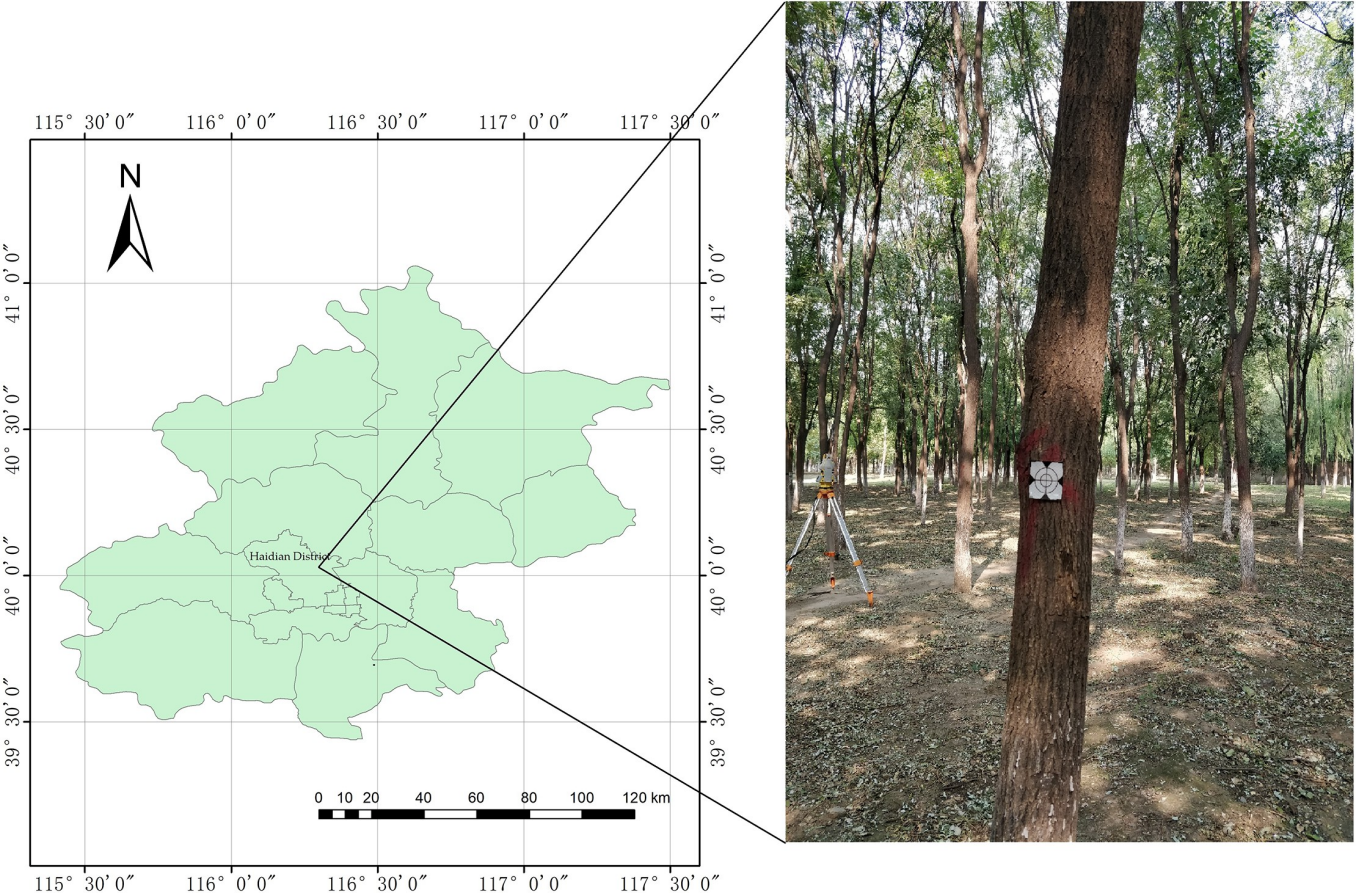
Study area in Haidian District, Beijing, China.

The study was carried out in an arbor forest sample plot located in Haidian District (116.20°E, 40.00°N), Beijing, China, in the summer of 2018. The dominant tree species in the study area is *Styphnolobium japonicum* (L.) SCHOTT. (syn. *Sophora japonica)*, followed by *Birch* and *Chinese pine*, and the sample plot had a stem density of approximately 1100 stems/ha as shown in Fig 1. The study area is a typical artificial woodland from northern China with sparse understory vegetation under the canopy, which is favorable for the scanner to obtain more accurate 3D spatial point cloud data of the targets with less noise.

### Ethics statement

The individual in this manuscript has given written informed consent (as outlined in PLOS consent form) to publish these case details.

### Experimental instrument

The ZEB-REVO-RT equipment developed by GeoSLAM Ltd. (UK) is a lightweight personal mobile laser-scanner (PLS), which consists of a laser scanner, a data logger, a camera, a low-cost IMU and accessories. It weighs approximately 3.5 kg. The laser scanner containing an eye-safe laser giving 43200 measurements per second is lightweight (1.0 kg) and small (86×113×287mm), making it more convenient for hand-held movement in forest surveys. With a maximum laser range of 30 m, the PLS is designed as an area scanner and works continuously for up to 4 hours. The ZEB-REVO-RT equipment can be used in a variety of ways, such as handheld, pole-mounted, or attached onto a mobile platform such as a vehicle or UAV. Instead of using GNSS within the navigation module, the PLS makes full use of simultaneous localization and mapping (SLAM) technology, which was developed by the machine vision and robotics community [22]. The concept of SLAM technology relies on the ability to place a robot at an unknown location in an unknown environment and then have it build a map, using only relative observations of the environment, and then to use this map to simultaneously navigate, which makes such a robot autonomous [38,39]. Currently, the SLAM technology has been widely used in large-scale mapping of urban structures [37], mine mapping [40], landslide investigation [41] and urban transport [42]. The problem of no GNSS signal or poor signal under the forest canopy can be solved by using PLS combined with SLAM technology in forest sample plot surveys.

The PLS allows several smartphones or tablets to seamlessly connect to the scanner via the Wi-Fi interface of the device, which enables the field data to be collected in the mobile terminal, allowing for real-time data visualization and eliminating the need for post-processing. Along with the direction of the investigator's movement, the PLS equipment captures a strip of the point cloud data at the speed of movement and generates a movement trajectory of the surveyor [43]. The manufacturer claims a measurement range of up to 30 meters. However, in practical field investigation, the measurement range of the ZEB-REVO-RT equipment is reduced to 15–20 m due to the influence of solar radiation [25]. The scanner performs a 270×360° auto-rotation scan in space during the process of measurement, enabling fast and easy 3D mapping without loss of detail. The manufacturer stated that the relative accuracy of measurement is±15 mm within 30 m [22]. Table 1 shows the detailed technical specifications of the ZEB-REVO-RT equipment.

**Table 1:** Technical specifications of the ZEB-REVO-RT.

Parameter	Value
**Laser measurement principle**	Time of flight
**Data acquisition speed**	43200points/sec
**Maximum range**	30 m (15m outdoors)
**Absolute position accuracy**	3-30cm
**Field of view angle**	270×360°
**Scanner line speed**	100Hz
**Measurement accuracy**	±15mm
**Wavelength**	905nm
**Battery life**	4h
**Camera**	GoPro
**Size**	86×113×287mm
**Total weight**	3.5kg

### Data acquisition

Tree position and DBH data were acquired from the field data and PLS point cloud data. The data acquired by field measurements served as the reference measurements, and the data derived from the PLS point cloud served as measurement values.

#### Field data acquisition

The field data were collected in a 300 m^2^ (15×20m) rectangular study area in early July of 2018. The collected data encompassed the species, DBH and locations of all the trees within the sample plot. The DBH of each tree of more than 5 centimeters was manually measured by a steel tape to the nearest millimeter at DBH height (1.3 m vertical above the ground from the base of the tree). The position information of each tree (X, Y, Z coordinate values) was recorded using the independent coordinate system established by the total station. In order to convert the coordinate system established by the total station into the coordinate system established by ZEB-REVO-RT equipment, the four marker plates were used to attach to four different tree trunks (the center of the four marker plates was not present in any plane of space at the same time). The total station was used to record the spatial coordinate information of the centers of the four marker plates.

#### PLS (ZEB-REVO-RT) data acquisition

**Fig 2:**
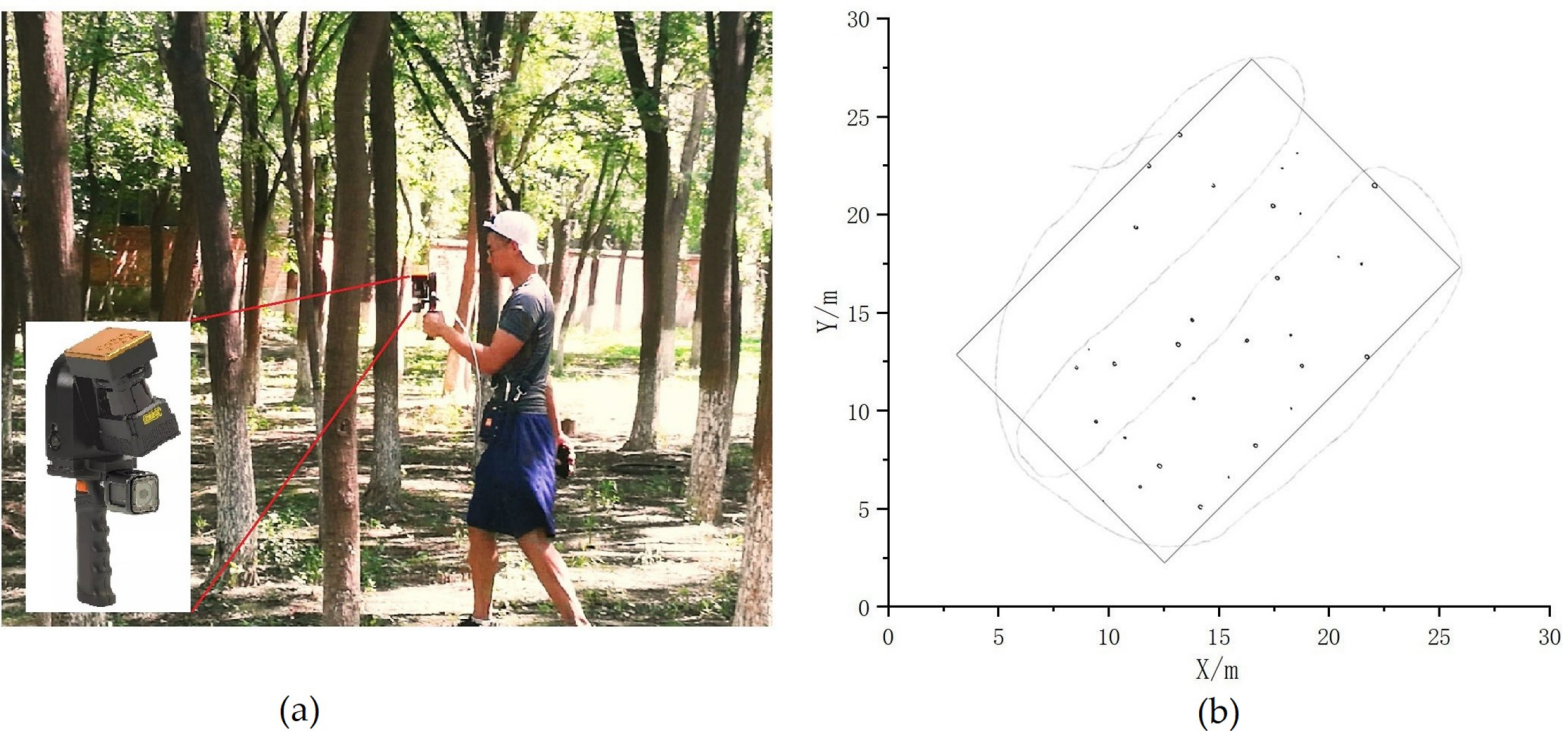
Field data collection and roadmap. (a) Data acquisition in the field using the ZEB-REVO-RT device; (b) the dotted line refers to the field scan path and spatial distribution of tree trunk point cloud data within the sample plot in the black point.

To ensure maximum coverage of all trees and acquire high resolution data in the test area, we used a method of serpentine scanning and walked slowly to acquire the point cloud, as shown in Fig 2(B). The setting of the scanning path mainly considers the following four main factors: (1) a suitable data acquisition path; (2) avoiding the occlusion problem among trees, and guaranteeing a better scanning coverage for the trees in the study area; (3) avoiding the problems associated with drift, which can arise once the SLAM algorithm fails to execute the alignment correctly; (4) reducing the scanning range noise to generate reliable point cloud data.

The ZEB-REVO-RT equipment firstly needs to initialize the IMU for establishing the reference coordinate system before the data acquisition starts. After approximately 15 seconds of initialization, the survey is executed by moving at walking speed whilst gently oscillating the ZEB-REVO-RT laser head forward and backward to capture data from the full 3D environment. The survey path should form a closed loop, so that the same region is covered at the beginning and the end of the path. The IMU integrated into the scanning head is capable of measuring angular velocities and linear accelerations that can be used to calculate the sensor’s trajectory [36]. The SLAM algorithm used to calculate the position of laser data adopts a linearized model to minimize the error in the IMU, and then generate reliable position and orientation values.

The recording of the entire plot (15×20m) takes approximately 5 minutes. The survey data will be processed automatically within the ZEB-REVO-RT equipment while the data are being collected. Furthermore, since registration is conducted in real time, the results are available almost immediately on completion of the survey. The real-time local processing service offered by GeoSLAM Ltd. can be used to check the survey data instantly on site using a smartphone or tablet. Real-time feedback enables us to see exactly what we have and haven't captured before the survey has even finished so nothing is missed. The ZEB-REVO-RT field measurement is shown in Fig 2(A).

### Pre-processing of point cloud data

When the field data collection is completed, the data logger processes the collected data in real time and generates a scan point cloud, which is displayed on a device such as a mobile phone or a tablet. The point cloud data generated by the ZEB-REVO-RT equipment is more accurate in a small range of acquisition areas. However, if the area is very large and the characteristics of the area are not distinctive enough, the accuracy of the SLAM algorithm will decrease, and the displayed result will be misaligned. In order to obtain more accurate processing results, it is necessary to re-process the collected scan data using the GeoSLAM desktop software which is an all-in-one solution for 3D point cloud manipulation.

**Fig 3:**
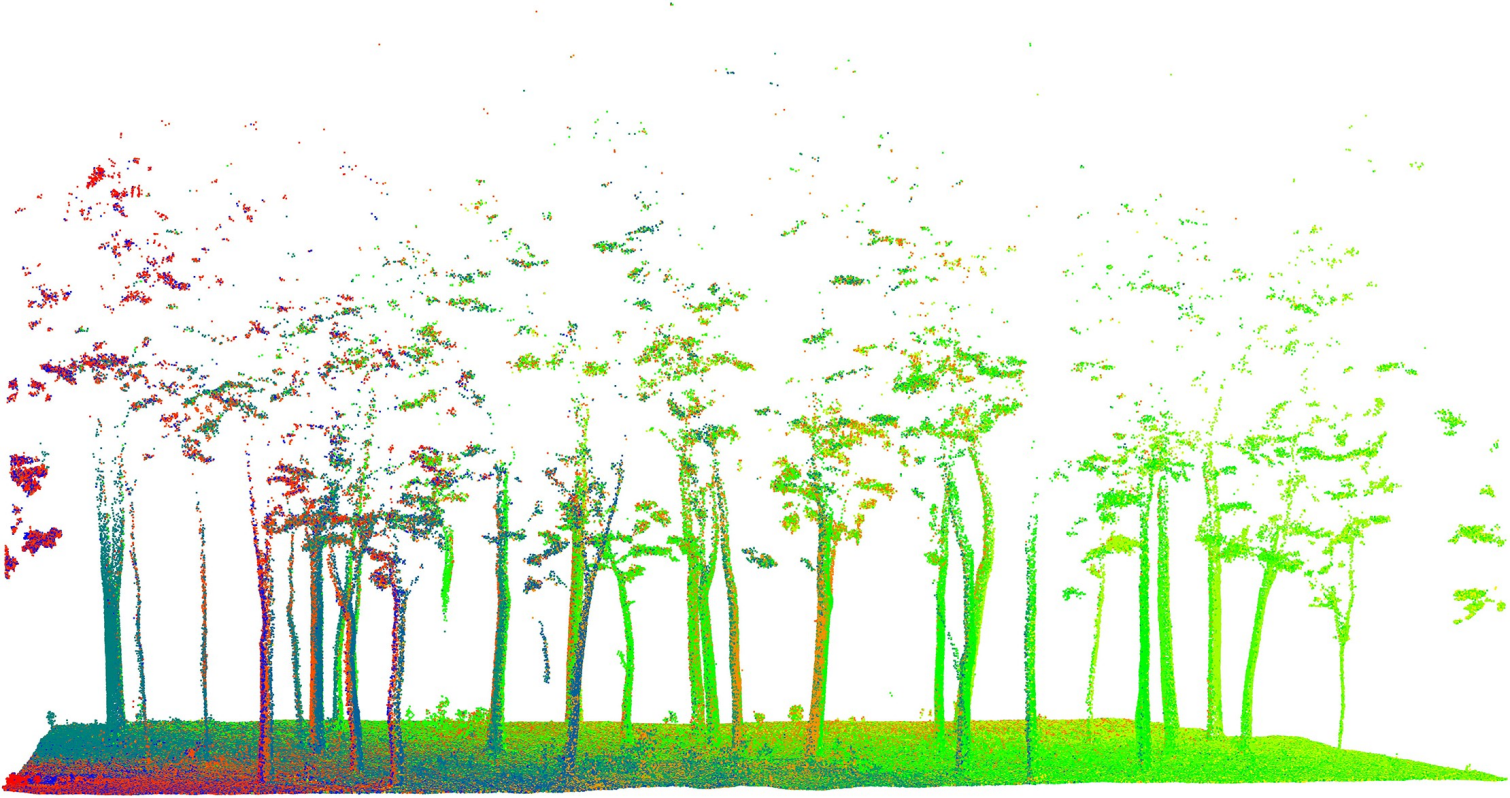
Pre-processed point cloud data.

Due to the influence of tree branches and understory vegetation during data collection, filtering represents a crucial part of point cloud data preprocessing. We selected the sample plot and used CloudCompare software to further process the point cloud data within the sample plot. The hybrid filtering de-noising method was adopted as a noise filter. In this method, the sphere radius value was set to 0.0466 m, and its function was roughly the same as the radius filtering. The default value of 1 was selected for the maximum relative error, and the method for removing outliers was similar to that of statistical filtering. Fig 3 shows the laser point preprocessed by CloudCompare software.

### Processing and modelling of point cloud data

In order to model the tree trunks precisely, we used LiDAR 360 software to further process the point cloud data. Firstly, we performed secondary denoising on point cloud data to eliminate the effects of outliers and other types of noise. Then, we filtered the point cloud data and generated DEM using the filtered ground points. Finally, the point cloud data were normalized to eliminate the influence of topographic fluctuations. Due to the presence of low-density point cloud data acquired by ZEB-REVO-RT equipment and the defects in the point cloud matching generated using the SLAM algorithm, the obtained DBH values may have a large deviation if the intercepted point cloud slice is directly fitted with circles or cylinders. Therefore, we proposed a method for solving the DBH values by calculating the polygonal cylindrical volume. The specific process was as follows: (1) We intercepted the point cloud data at 1.2 m to 1.4 m and 1.1 m to 1.5 m at the trunk (the trees in the sample plot had no branches at 2.0 m and below.), respectively, and then used the point cloud data fitting polygonal cylinder. (2) Calculating the volume of the fitted polygonal cylinder; (3) Calculating the DBH values of the trees using the cylindrical volume formula (1).

$$V=\frac{\pi D^{2}}{4}h$$ (1)

where **V** is the volume of the fitted polygonal cylinder, ***D*** is the diameter of the fitted cylinder, ***h*** is the height of the horizontal slice.

**Fig 4:**
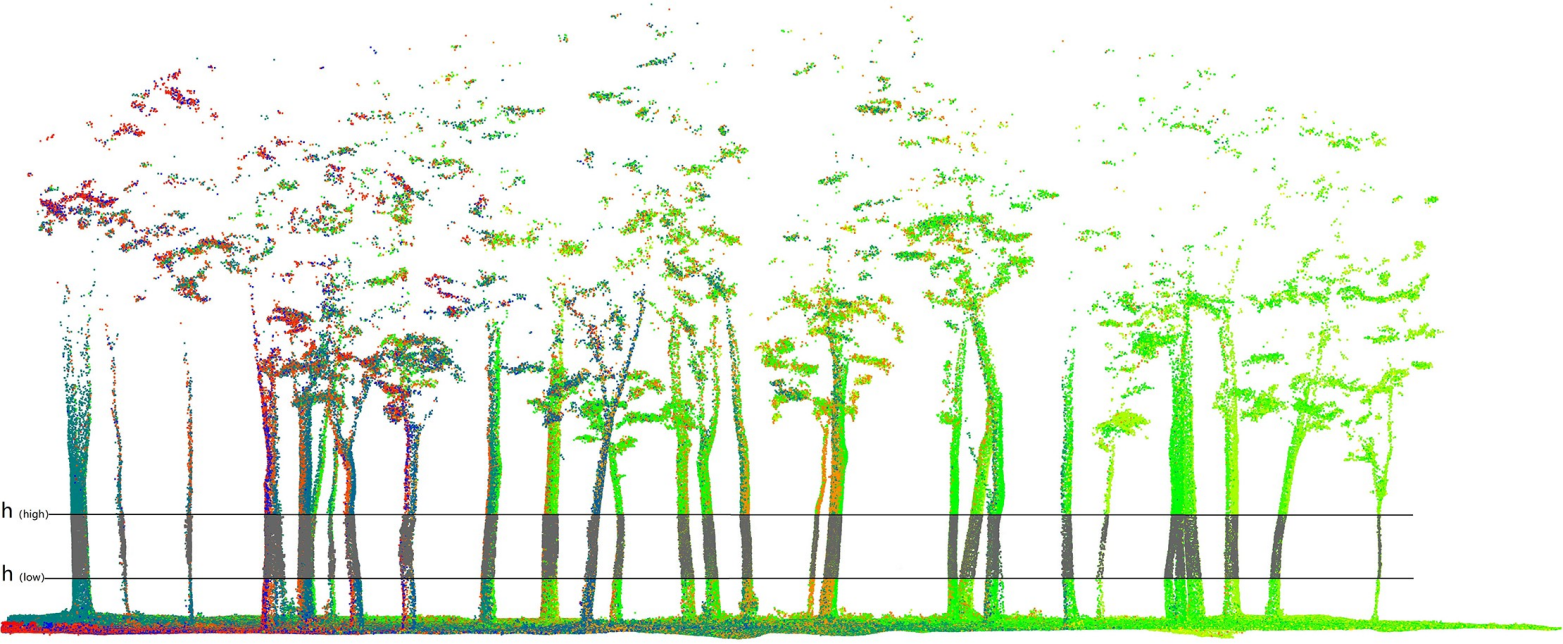
Strip on the normalized point cloud. The gray points in the strip are used to fit the cylinder. (***h***_(***high***)_ is 1.5 meter and 1.4 meter respectively, corresponding to ***h***_(***low***)_ of 1.1 meter and 1.2 meter).

The diameter of the trees in the sample plot was analyzed based on the trunk in the horizontal strip. Two parts (i.e. 1.2–1.4 m and 1.1–1.5 m) of point cloud data were collected from the tree trunk point cloud as the strip (shown in Fig 4), after which the points between the ***h***_(***high***)_ cross section and ***h***_(***low***)_ cross section were fitted with the cylinder. The definition of the value of the upper cross-section of the strip avoids the influence of the point cloud generated by the branches and the upper canopy on the fitted cylinder. At the same time, the definition of the value of the lower cross-section avoids the influence of the point cloud generated by the surface vegetation and low shrubs on the fitted cylinder.

Before the cylinder modeling of point cloud data, we need to individualize the point cloud of the trunk within the strip, and finely model the segmented point cloud one by one, which helps us timely check which point cloud of the trunk is not successfully modeled and assess the existing problems. To ensure the convergence of the point cloud data iteration, and that the point cloud data is successfully modeled, the point cloud data used in our modeling must be normalized.

We approximately considered that the part of the trunk below 1.5 m is vertical, and that the central points of the individual trunk point cloud data in the strip 1.25–1.35 m is the position of the tree. The trunk points that belong to the same tree were clustered based on location and distance analysis. All points less than 0.3 m apart were clustered into a group of points and were considered as belonging to the same tree. Points that were more than 0.3 meters away were considered to belong to different trees. The reason for choosing 0.3 meters is that all the trees in the sample plot had a breast diameter of less than 30 cm. Therefore, using such an approach helps uniquely identify each tree. The center position of each point group was calculated and determined.

### Evaluation of the accuracy of the mapping results

To evaluate the accuracy of the mapping results, the reference data measured in the field was compared with the PLS (ZEB-REVO-RT) data. The criteria for the mapping results included omission errors, commission errors, trunk detection accuracy and tree location accuracy. Omission errors refer to trees that exist in the sample plot, but were not accurately detected in the point cloud data acquired by the ZEB-REVO-RT equipment. Commission errors were defined as the trunk models mapped from point cloud data for which no corresponding trees were found in the sample plot. The trunk detection accuracy was the percentage of trees in the plot that were correctly detected. The tree location accuracy was the degree of deviation between the tree location in the mapping and the corresponding tree location in the sample plot, which was reflected by bias and root mean squared errors (RMSE). Table 2 shows the accuracy requirements of different investigation factors at different survey levels in China's forest inventory.

**Table 2:** Permissible error rating table of main investigation factors in forest inventory [44].

Investigation factor	A-level error	B-level error	C-level error
**Sub-compartment area**	5	5	5
**Tree species composition**	5	10	20
**Tree height**	5	10	15
**DBH**	5	10	15
**Age**	10	15	20
**Canopy density**	5	10	15
**Sectional area per hectare**	5	10	15
**Volume per hectare**	15	20	25
**Number of trees per hectare**	5	10	15

The bias, root mean squared error (RMSE), relative bias and relative RMSE were employed to gauge the accuracy of the DBH estimations, as defined in the following equations:

$$Bias=\frac{1}{n}\sum_{i=1}^{n}e_{i}=\frac{1}{n}\sum_{i=1}^{n}\left(y_{i}-y_{j}\right)$$ (2)

$$Bias\%=\frac{Bias}{\bar{y_{j}}}\times 100\%$$ (3)

$$RMSE=\sqrt{\frac{\sum_{1}^{n}{\left(y_{i}-y_{j}\right)}^{2}}{n}}$$ (4)

$$RMSE\%=\frac{RMSE}{\bar{y_{J}}}\times 100\%$$ (5)

where *y*_*i*_ is the ith measurement value, *y*_*j*_ is the jth reference value, $\bar{y_{j}}$ is the mean of the reference values, and n is the number of estimations.

## Results

### Trunk mapping results

The data on the trunks detected and mapped using ZEB-REVO-RT equipment are listed in Table 3. The total number of trees in the sample plot was 33, all of which were *Sophora japonica*, and their diameters were distributed between 5.1 cm and 21.5 cm. The point cloud dataset obtained using the ZEB-REVO-RT equipment was used to successfully model 30 strunks, with a detection accuracy of 90.9%. The correct detection rate of the trunks reached 93.3% without considering the measurement errors at the border of the plot.

**Table 3:** Accuracy of trunk mapping using the ZEB-REVO-RT data.

	Species	Reference	Mapped	Omission	Commission
**Number of trunks**	Sophora japonica	33	30	3	2
**Percentage (%)**	\	100	90.9	9.1	6.1

Three trunks that were not mapped accounted for 9.1% of the total. One tree was cut off when the sample plot was split because it was located on the boundary of the sample plot. The missed trees in the interior of the sample plot were mistaken for noise points and filtered out due to the thinness of their trunks and partial occlusion of the trees. The commission errors of two trunks that were located on the border of the sample plot accounted for 6.1% of the total.

### DBH estimation

**Fig 5:**
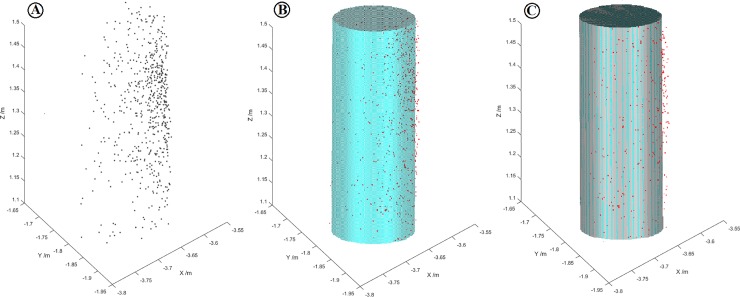
The fitted cylinders of non-contact features in the strip of 1.1–1.5m. (A) Diagram of the trunk points in three-dimensional coordinates; (B) cylinder fitted by trunk points; (C) fitted polygonal cylinder composed of a large number of triangles.

**Fig 6:**
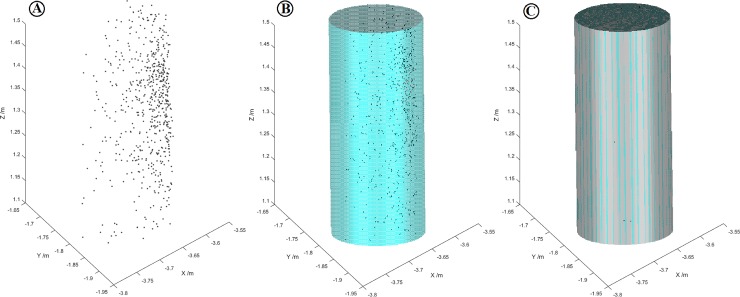
The fitted cylinders of contact features in the strip of 1.1–1.5m.

The individual trunks were modeled within the strip (Fig 4) utilizing the Geomagic software. By keeping 100% sampling of the tree trunk point data, the characteristic information of the tree trunks was maintained to the maximum extent. In order to obtain a more accurate fitting diameter, the fitted cylinder of contact feature and the fitted cylinder of non-contact feature were respectively used on the tree trunk points. The polygonal transformation of the fitted cylinder and the features of the tree trunk points were used to create a new cylinder composed of a large number of triangles (e.g. the cylinder in Fig 5C was composed of 8720 triangles with a maximum deviation of 0.25 mm; the one in Fig 6C was composed of 9426 triangles with a maximum deviation of 0.27 mm). Subsequently, the volume of the polygonal cylinder was calculated and the diameter of the trunk was obtained.

In the strip of 1.1–1.5 m, the standard deviation of the polygonal cylinder fitted by the non-contact features of all trunk points was in the range of 0.66–1.21 cm, among which the standard deviations of less than 1 cm accounted for about 80% of the total deviation. The shape deviation was between 3.80–11.00 cm, among which the shape deviations of less than 8 cm accounted for about 90% of the total deviation. The standard deviation of the polygonal cylinder fitted by the contact features of all the trunk points was in the range of 0.77–1.24 cm, and the shape deviation was between 3.80 cm and 10.85 cm. In the strip of 1.2–1.4 m, the standard deviation of the polygonal cylinder fitted with the non-contact features of the tree trunk point was within the range of 0.64–1.27 cm, among which the deviations of less than 1 cm accounted for 86.7% of the total deviation, and the shape deviation was between 2.76–11.06 cm, among which the shape deviations of less than 8 cm accounted for about 96.7% of the total deviation. The standard deviation of the fitted cylinder corresponding to the contact features was between 0.68 cm and 1.4 cm, and the shape deviation varied from 3.06 cm to 11.06 cm. The results show that the standard deviation and shape deviation of the polygonal cylinders obtained by different fitting methods of different strips was similar.

Table 4 lists the results of DBH estimation from non-contact fitted cylinders, indicating that the DBH values for the same trunks fitted in the two strips (i.e. 1.2–1.4 m and 1.1–1.5 m) were similar. The reference measurements of the DBH were distributed in the range of 5.9–21.5 cm, and in the strip of 1.1–1.5 m, the DBH values obtained by fitting the cylinder volumes were distributed between 4.6–20.1 cm. In the strip of 1.2–1.4 m, the values of the DBH obtained by fitting the cylinder volume were distributed in the range of 5.1–20.1 cm.

**Table 4:** The accuracy of the DBH from non-contact fitted cylinders.

	Strip	Bias(cm)	Bias (%)	RMSE(cm)	RMSE (%)
**DBH**	1.2m-1.4m	-1.19	-8.98	1.62	12.23
**DBH**	1.1m-1.5m	-1.26	-9.68	1.58	11.88

Table 4 shows that the overall deviation of the DBH estimation of the fitted tree trunks in the 1.2–1.4 m strip was -1.19 cm and the RMSE of the DBH estimation was 1.62 cm. By contrast, in the 1.1–1.5 m strip, the overall deviation of the DBH estimation of the fitted tree trunks was -1.26 cm and the RMSE was 1.58 cm. The relative deviations (Bias %) of the DBH estimations in these two strips were -8.98% and -9.68%, respectively, and the relative root mean square errors (RMSE %) were 12.23% and 11.88%, which met the C-level error requirements for China's forest inventory regulations.

The accuracy of the DBH estimation from contact fitted cylinders is listed in Table 5. It can be seen that the DBH obtained by fitting the cylinder volumes in different strips was basically consistent with the overall deviation and RMSE, but it was significantly different from the reference of the DBH measured in the field. The overall deviation and RMSE of the DBH estimations of the trunks fitted in the strips 1.2–1.4 m were 2.50 cm and 3.27 cm, respectively. The overall deviation and RMSE of the DBH estimations of the trunks fitted in the strips at 1.1–1.5 m were 2.60 cm and 3.27 cm, respectively. The relative deviations (Bias %) of the DBH estimations in these two strips were 18.82% and 19.60%, respectively, and the relative root mean square errors (RMSE %) were 24.60% and 24.63%, respectively, which did not meet the error requirements of the forest inventory regulations in China.

**Table 5:** The accuracy of the DBH from contact fitted cylinders.

	Strip	Bias(cm)	Bias (%)	RMSE(cm)	RMSE (%)
**DBH**	1.2m-1.4m	2.50	18.82	3.27	24.60
**DBH**	1.1m-1.5m	2.60	19.60	3.27	24.63

### Estimation of tree positions

**Fig 7:**
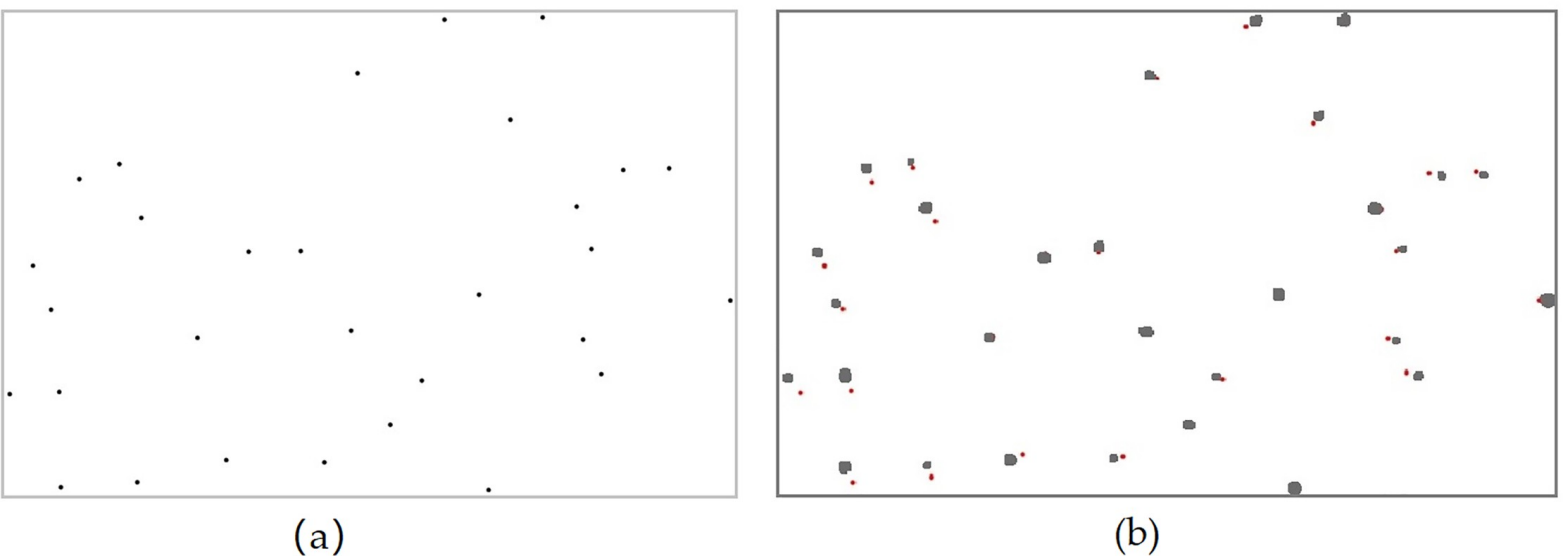
Estimation of tree positions. (a) Positions of trees observed using the total station. (b) Positions of trees obtained using ZEB-REVO-RT compared with their observed positions (red dots indicate observed tree locations, gray dots indicate the position of the trees obtained using the ZEB-REVO-RT equipment).

The tree position data in the total station coordinate system was converted to the ZEB-REVO-RT coordinate system via the marker points. The center position of each point group was calculated, and the distance from the center of each tree to the tree position measured using the total station was manually measured in the software. The spatial distribution of trees in the field assessed using the total station is shown in Fig 7(A), and a comparison of the positions of the trees obtained by the ZEB-REVO-RE equipment with the observed positions of the trees is shown in Fig 7(B). A comparison of the measured values of the tree positions with the reference measurements is summarized in Table 6.

**Table 6:** Evaluation of the accuracy of the estimation of tree positions.

	Strip	Bias(m)	RMSE(m)
**Position**	1.1m-1.5m	0.24	0.26

### Comparison of the efficiency of PLS survey and field survey methods

The survey time was recorded to analyze and compare the efficiency of the PLS survey and field survey methods. Both survey methods were applied to the same sample plot (15×20m). The ZEB-REVO-RT survey time, which includes data collection and data processing, was approximately 10 minutes. The data collection time includes the time spent in automatic calibration at the beginning of data collection and automatic registration at the end. The field survey time mainly includes recording the DBH values, tree species and determining the tree locations. At least three investigators are involved in the field survey, while only one is needed in the ZEB-REVO-RT equipment survey. In addition, the data collected by the PLS instruments requires additional processing to obtain the DBH and tree locations, while the field survey method can yield these parameters at the end of the field survey. The time spent on the two survey methods is shown in Table 7.

**Table 7:** Comparison of the efficiency of the two survey methods.

Survey Method	Personnel	Area (m^2^)	Consuming time (min)	Survey coverage per surveyor (m^2^/min)
**PLS(ZEB-REVO-RT)**	1	300	10 (survey (5) + processing (5))	30
**Field survey**	3	300	110	0.91

### Analysis of the mapping results of the PLS data

**Fig 8:**
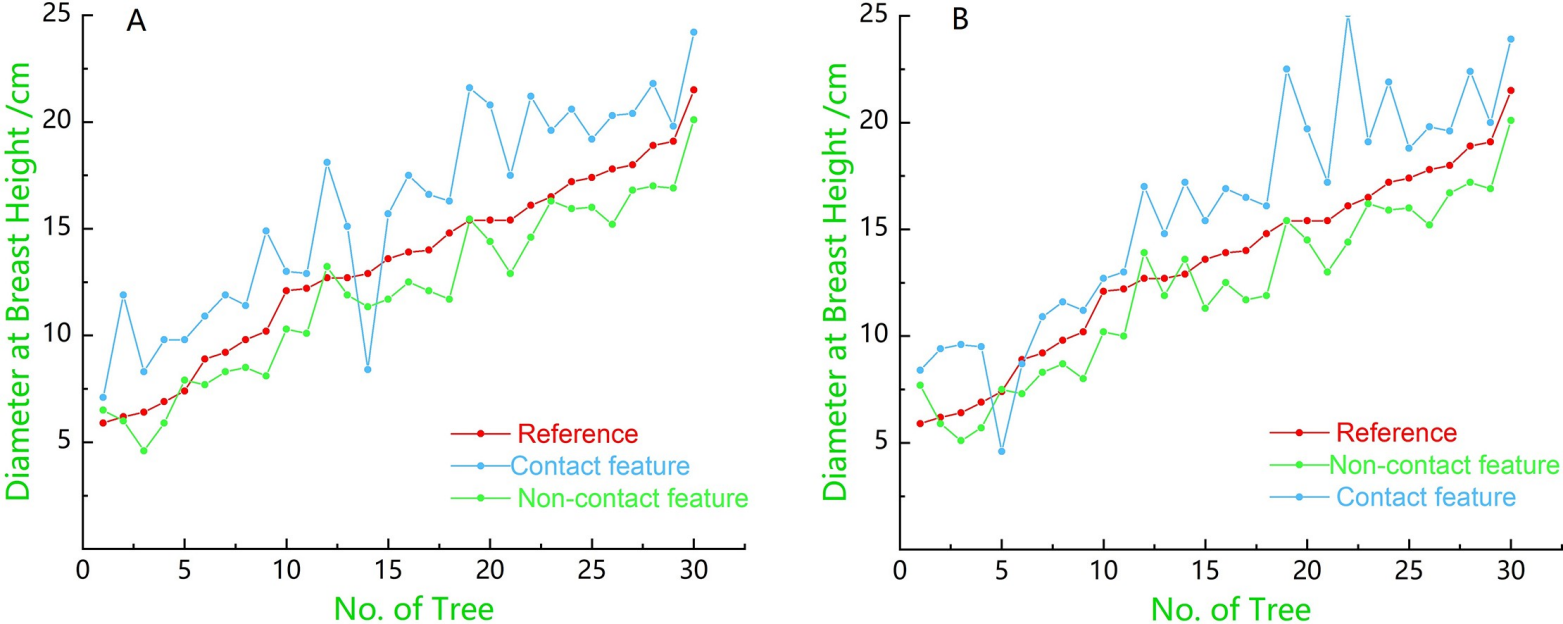
Comparison of the trunk DBH estimates in the two strips with the reference measurements. (A) Comparison of the DBH values of the two differently fitted cylinders in the strip at 1.1–1.5 m with the reference measurements; (B) Comparison of the DBH values of the two differently fitted cylinders in the strip at 1.2–1.4 m with the reference measurements. Reference refers to the field measurements; Contact feature refers to the DBH values obtained via the fitted polygonal cylinder of contact features; Non-contact feature refers to the DBH values obtained via the fitted polygonal cylinder of non-contact features.

The point cloud data of the target in the sample plot were obtained using the ZEB-REVO-RT instrument. They were used to extract the DBH and position information of the trunks. According to the data of the tree trunk points, the methods of fitting the polygonal cylinders with contact features and non-contact features were used. Fig 8 shows a line graph of the DBH values obtained from the fitted cylinders and the DBH values ​​measured in two different strips in the field. It can be seen that the DBH values obtained using the fitted polygonal cylinders with contact features significantly overestimated the measured DBH values. This is because when using this method to fit the trunk point data, the farthest point in the point cloud data in the same plane is used as the boundary of the polygonal cylinder. In Fig 8A, there is a value in the DBH obtained using the fitted cylinder of the contact feature that is smaller than the corresponding reference value. It was found that the point cloud data acquired using the ZEB-REVO-RT instrument only yielded the relative point in one direction of the trunk, and the number was small. In the process of filtering, a large part of the trunk point data was filtered out, which resulted in a smaller fitting of the polygonal cylinder than the actual trunk when fitting the cylinder with the contact features. Fig 8B shows a similar situation.

**Fig 9:**
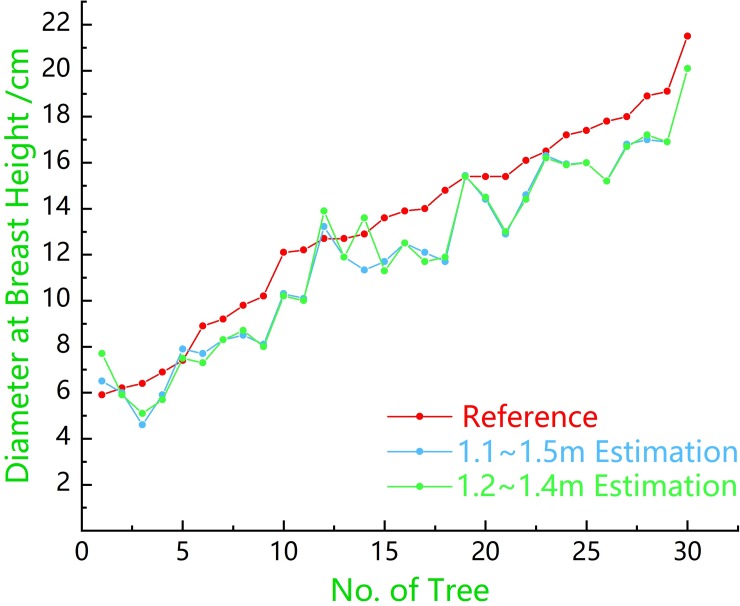
Comparison of the DBH estimations of non-contact features with reference values. Reference refers to the field measurements; 1.1–1.5 m estimation refers to the DBH estimations of the 1.1–1.5 m part of the trunk; 1.2–1.4 m estimation refers to the DBH estimations of the 1.2–1.4 m part of the trunk.

From Fig 9, we can see that the DBH estimations obtained by fitting the polygonal cylinder with the point cloud data of the two strips were similar, which significantly underestimated the reference measurements. We can preliminarily draw the conclusion that the DBH estimations of the trunk are similar by horizontally intercepting the point cloud data of different thickness (i.e., 20 cm and 40 cm) at the chest diameter of the trunk to fit the polygonal cylinders. The deviations of the DBH estimations were -1.26 cm and -1.99 cm, respectively, while the corresponding root mean square errors (RMSE) were 1.58 cm and 1.62 cm, respectively. The DBH estimates for the fitted polygonal cylinders obtained for different trunk diameters were not significantly different from the measured reference values, since in this study, the reference measurements were within the range of 5.9–21.5 cm.

**Fig 10:**
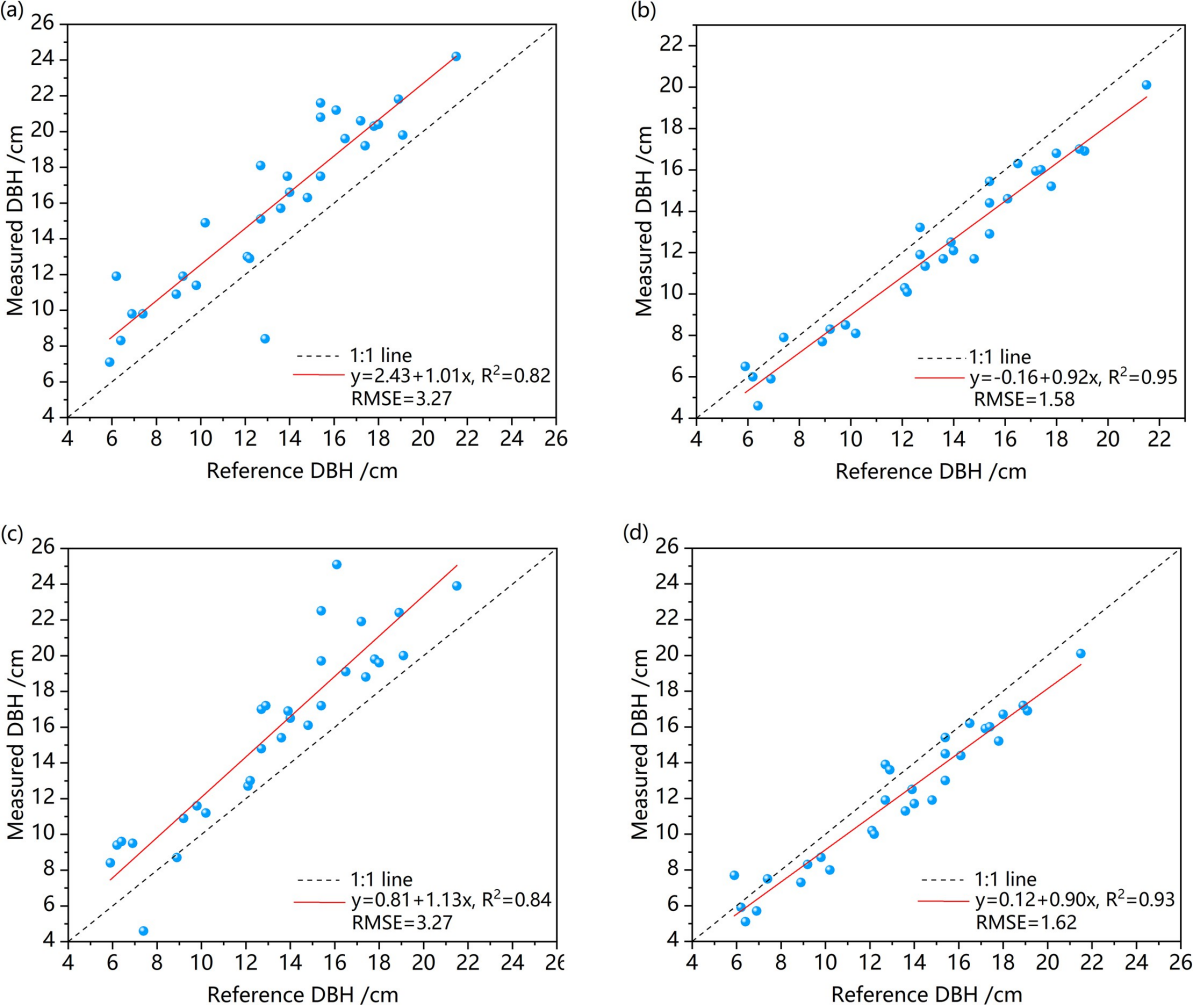
Scatterplots of the DBH measured with a steel tape versus the DBH derived from different strips with different fitting methods. The dashed line shows the 1:1 line and the red solid line shows the trend-line. (a) and (b) represent the DBHs estimations of contact features and non-contact features in the strip 1.1–1.5m respectively; (c) and (d) represent the DBHs estimations of contact features and non-contact features in the strip 1.2–1.4m respectively.

As can be seen from the different fitting methods, the trunk diameters obtained by fitting the polygonal cylinders using the non-contact features significantly underestimated the reference measurements, which were closer to the measured diameters of the trunks than those obtained using the fitting method of contact features. Fig 10 shows a comparison of the DBH estimations and the reference measurements. The comparison of the DBH values estimates and the reference DBH values revealed that the diameter accuracy of the fitted cylinder using the non-contact features in the 1.1–1.5 m strip is optimal.

The DBH values estimations obtained by the volume of the fitted polygonal cylinder of non-contact features were closer to the reference measurements than those obtained using contact features. The non-contact features method for fitting the polygonal cylinders was more robust, while the contact features method as poorer in performance and was highly susceptible to noise, which directly caused a large error in the estimations of the breast diameters.

## Discussion

In this study, a method for calculating the DBH via the volume of a fitted polygonal cylinder is proposed, which is similar to the method of fitting a cylinder. The DBH values of the trunks obtained by this method were not significantly different from the reference values of the DBH values measured in the field. PLS (ZEB-REVO-RT) instruments offer unique investigative advantages such as the ability to quickly acquire 3D point cloud data without GNSS signals for positioning, which offers great promise for practical applications in forest surveys. However, there are still some issues in the processing and mapping results of the point cloud data [43, 45,46].

### Removing noise

**Fig 11:**
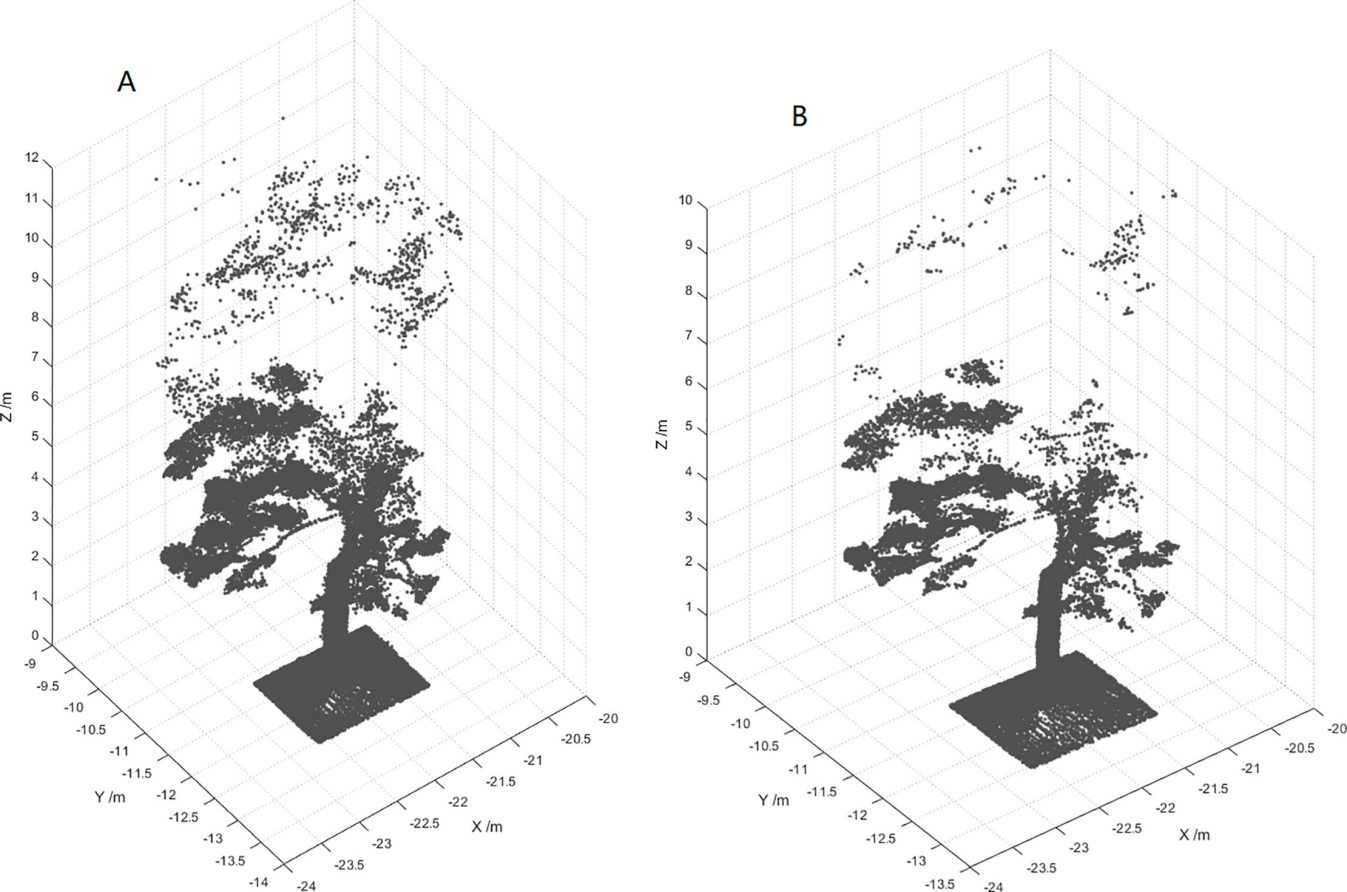
Point cloud of a single tree. (A) The first filtered tree trunk point cloud. (B) Secondary filtered tree trunk point cloud.

There was significant noise in the point cloud data collected by the ZEB-REVO-RT equipment [43, 45]. After the first filtering, there was still noise in the obtained point cloud data (Fig 11A). To further reduce the influence of noise on the fitted trunk diameters, secondary filtering of the point cloud data with optimal threshold parameters was conducted to improve the accuracy of the DBH estimations. We performed filtering tests many times by setting different thresholds and compared the test results. Finally, we set the number of domain points to 10 and the standard deviation to 5 times for secondary filtering. The point cloud data after the secondary filtering had less noise (as shown in Fig 11B) and was used for further processing.

### Comparison of measurement results

According to previous studies, we know that ALS, TLS, MLS and emerging PLS are affected by tree density in the field. The smaller the density of trees in the sample plot, the higher the detection rate of the trunks, and the greater the density of the trees, and the lower the detection rate. In sparse forests with a trunk density of 100–200 stems/ha, the detection range of the stems can reach 70% to 100%. In a forest with a trunk density of more than 1000 stems/ha, the trunk detection rate is approximately 70% [19]. Maas *et al*. [17]investigated four plots with a trunk density of 212–410 stems/ha, and the detection rate of the trunks was in the range of 86.7% to 100%. In Carlos Cabo *et al*. [47], in three plots with densities of 300 stems/ha, 900 stems/ha and 2100 stems/ha, the completeness of trunk detection was 100% and the correctness of trunk detection was 98.5–100%. In addition, the detection rate of the trees was also affected by the laser scanning mode. In the study by Lovell *et al*. [48], with single-scan (SS) method using TLS in the plots with densities of 124 stems/ha and 477 stems/ha, the average detection rate of trees reached 54% and 68%, respectively. In the study by Liu *et al*. [49], three different terrestrial laser scanning methods (single center scan, MSS method, matched multiple scans) were used in 10 plots with trunk densities of 340–1200 stems/ha. The mean completeness and correctness were 70% and 94.2%, 80.8% and 90.9%, as well as 73.1% and 97.2%, respectively. In this study, if the measurement error of trees located on the boundary of the plot is not taken into account, the correct detection rate of the trunks using ZEB-REVO-RT equipment is 93.3% in the sample plot with a density of 1100 stems/ha. Compared with the study by Liang *et al*. [14], which used PLS instruments to achieve an overall trunk detection rate of 82.6%, the tree detection rate in this study was higher.

The diameter at breast height (DBH) was assessed by comparison with field-measured breast diameter results. This comparison was performed using different fitting methods for point cloud slices of different thicknesses. By different fitting methods, the optimal fitting results of deviation and root mean square error of -1.26 cm and 1.58 cm were obtained. In the available literature, the breast diameters obtained by different terrestrial laser scanning methods were also different. Liu *et al*. [49] used the SS, MS and MSS methods to obtain tree parameters in 10 sample plots. For the SS method, the MS method, and the MSS method, the deviations and the RMSE of the DBH estimation were in the range of -0.30–1.01 cm and 1.1–3.07 cm, 0.18–1.75 cm and 0.73–2.70 cm, as well as -0.04–0.66 cm and 0.96–4.28 cm, respectively. Liang *et al*. [14] used PLS instruments to map the trunks of the sample plot, and arrived at an RMSE of the DBH values estimation of 5.06 cm, which was significantly lower than the measurement accuracy in this study (1.58 cm). In the study by Joseph Ryding *et al*. [22], the results indicated that there was a significant difference in the fitting accuracy of the trunks with diameters greater than 10 cm and less than 10 cm. When the DBH was greater than 10 cm, the overall deviation and the RMSE were 0.9 cm and 1.5 cm, respectively. By contrast, the overall deviation and the RMSE when the DBH was less than 10 cm were 1.6 cm and 3.9 cm. However, the reference measurements of the DBH values of the trees in this study were between 5 cm and 22 cm, and the deviation of the DBH estimations was not significantly different in the range of the reference DBH greater than 10 cm and less than 10 cm. The effect of diameter size on mapping accuracy needs to be studied further.

### The advantages and challenges of PLS

The PLS (ZEB-REVO-RT) instrument has higher mobility than ALS, TLS or MLS, and it can be used in areas where the mentioned three methods are not applicable. Although airborne laser scanning (ALS) is less restricted by topographical factors and has high efficiency for large-area forest surveys, the acquired tree parameters and tree canopy structure information are limited, especially the trunk diameters and tree heights. Both TLS and MLS are greatly restricted by terrain factors. They are not applicable in some forest conditions with large topographic fluctuations and steep terrains, and the PLS may be a very good choice in such cases. Only one operator can implement all the data collection work using the ZEB-REVO-RT equipment. The data from the surrounding environment is rapidly acquired by constant scanning when the operator moves forward, and a synchronous video can be observed in the data collection process, saving a lot of time compared to TLS and field data collection [22]. Hence, it is more efficient than the field investigation. When the data collection is completed, the collected point cloud data can be viewed and checked in the field through a smartphone or tablet to ensure the integrity of the collected data, and the density and quality of the collected point cloud data can be checked on-site. The ZEB-REVO-RT equipment automatically processes the collected data in real time through an online processing service provided by GeoSLAM Ltd., which reduces the processing time of the data. The PLS instruments combined with the SLAM algorithm have a higher registration accuracy in forest surveying than that of the MLS equipment, and can acquire and process point cloud data under the canopy in real time without GNSS signals, which can solve the positioning problem in areas without a GNSS signal or weak GNSS signal under the forest canopy. This again reduces the processing time.

The automatic co-registration of PLS (ZEB-REVO-RT) equipment would fail if the sample plots had a lower or higher stem density, especially in case of a dense understory with moving leaves. The lower stem densities hindered the object recognition in the SLAM algorithm, leading to a “slip” of the object recognition algorithm. A higher stem density and dense understory would affect the co-registration of the ZEB-REVO-RT scan, which resulted in slight offsets of the point at the stems and presence of many double stems in the point cloud. Due to the limitations of the ZEB-REVO-RT’s equipment measurement range and the effects of ambient solar radiation, the farther the measurement distance is, the lower the point density of the laser. This limits the measurement of tree heights. A laser scanner with a higher range and lower divergence is therefore needed to better determine tree heights. It is an important factor to assess the timber volume and biomass. When there is more vegetation or branches under the forest canopy, there will be more noise in the collected point cloud data. Thus, it is necessary to set an optimal threshold to filter the point cloud data. A single scan time using ZEB-REVO-RT equipment is recommended for no more than 20 minutes. With a single scan time of more than 20 minutes, there will inevitably be some drift in the collected point cloud data. To reduce the drift of point cloud data registration, the data collection route should form a closed loop by starting and ending the scan at the same point.

In this study, the ZEB-REVO-RT equipment was used to achieve a correct detection rate of 93.3% of the trunks within the sample plot. Through the optimal fitting of the non-contact features, the deviation and the RMSE of the DBH estimations were -1.26 cm and 1.58 cm, respectively, which meets the C-level accuracy requirements of China’s forestry resources survey. Nevertheless, the accuracy of the DBH estimations may be significantly influenced by the quality of the point cloud acquired using the ZEB-REVO-RT combined a SLAM algorithm in the forest sample plots, as Bauwens *et al*. [25] noticed. The ZEB-REVO-RT equipment needs to be further studied to estimate the completeness and correctness of tree trunks in forest areas with different tree density and at different growth stages. The accuracy of the DBH estimations measured with ZEB-REVO-RT equipment for different tree diameters also needs to be further analyzed and studied. The error of tree positions obtained via point cloud data meets the requirements of precision in actual surveys in China, and the determination of tree positions is not affected by the GNSS signal. The survey method using ZEB-REVO-RT equipment shows that the survey coverage area per unit of time is higher than that of the field survey, which greatly reduces the time spent in the survey and provides a possibility for large-scale forest surveys. However, due to the limited measuring range and poor penetration of the ZEB-REVO-RT equipment, it is difficult to measure the top parts of the canopy in heavily occluded forests to obtain reliable tree height information. However, this is an important factor in the assessment of timber volume and biomass. At the same time, since the point cloud data of ZEB-REVO-RT does not include detailed canopy structure information of the upper part of the trees, the species and status information of the trees cannot be obtained from its point cloud data.

## Conclusions

In this study, personal laser scanning (PLS) equipment combined with SLAM technology was used to acquire the tree parameters in a forest sample plot (eliminating the influence of GNSS signals), and a method for calculating the DBH of the trees using a fitted polygonal cylinder volume was proposed. The point cloud data for slices of different thicknesses (i.e., 20 cm and 40 cm) was evaluated using different cylinder fitting methods (cylinder fitting of contact features and cylinder fitting of non-contact features), and the calculated DBH values of the trees obtained using the fitted cylinder of non-contact features was closer to the DBH measured manually using the steel tape. The same fitting method was used to fit the data of the slices of different thicknesses, and the measurement error between the calculated DBH values of the trunks and reference measurements was similar. The optimal breast diameter deviation and RMSE of the cylinders fitted using non-contact features were -1.26 cm and 1.58 cm, respectively, which meets the C-level accuracy requirements of China’s forestry inventory. The accuracy of the point cloud data obtained using the ZEB-REVO-RT equipment to detect the correct rate of the trunk, the accuracy of the DBH estimations and the tree positions were evaluated and analyzed. In term of survey time, the PLS instrument had a unique advantage. The trial showed that the PLS equipment has broad application prospects in forest inventory.

In this study, the advantages and limitations of PLS (ZEB-REVO-RT) equipment were analyzed in detail. The applicability of PLS equipment for forest areas with different densities and different growth stages needs to be further studied, as well as its use in complex topographies and areas with understory vegetation with different densities.

## Supporting information

S1 Table Field acquisition of diameter at breast height (DBH) and tree position data. (XLSX)

S2 Table The time taken to obtain the DBH and tree position information for both measurements (Time-consuming). (XLSX)

S1 File The point cloud data of the study area were obtained using personal laser scanning equipment. (LAS)

S1 Text Tree location information obtained using total station equipment. (TXT)
